# Efficacy of *Yang Yin Sheng Xue* formula against canine lymphoma chemotherapy-induced myelosuppression

**DOI:** 10.3389/fvets.2025.1635504

**Published:** 2025-08-12

**Authors:** Wanbing Pan, Ruoxi Sun, DengShan Shiau, Huisheng Xie, Jun Dong, Jiahao Lin

**Affiliations:** ^1^State Key Laboratory of Veterinary Public Health and Safety, College of Veterinary Medicine, China Agricultural University, Beijing, China; ^2^China Veterinary Medicine Innovation Center, China Agricultural University, Beijing, China; ^3^Department of Traditional Chinese Veterinary Medicine, Chi University, Reddick, FL, United States; ^4^China Agricultural University Veterinary Teaching Hospital, Beijing, China

**Keywords:** myelosuppression, *Yang Yin Sheng Xue* formula, chemotherapy, canine, adverse effects

## Abstract

**Introduction:**

Lymphoma is a prevalent malignant tumor in canines, with chemotherapy as the primary treatment approach. However, chemotherapy indiscriminately targets all rapidly dividing cells, including normal hematopoietic cells, leading to myelosuppression. Recent veterinary practices still lack standardized and effective management strategies for myelosuppression. This study aimed to evaluate a novel treatment strategy, utilizing the Yang Yin Sheng Xue formula (YYSXF), to alleviate chemotherapy-induced myelosuppression in canines with lymphoma.

**Methods:**

A mouse model of myelosuppression was established via intraperitoneal (i.p.) injection of cyclophosphamide (CP, 350 mg/kg). Different concentrations of YYSXF were administered, and peripheral blood cell counts were recorded. Bone marrow nucleated cells (BMNCs), hematopoietic stem cell (HSC) proportions, and apoptosis rates of bone marrow cells (BMCs) were determined. PI staining was performed to investigate YYSXF’s effect on cell cycle progression in the S and G2/M phases of BMCs. Histopathological changes in sternum bone marrow were examined through pathological sections. The outcomes of multicentric lymphoma in 11 canines treated with either CHOP chemotherapy alone or in combination with YYSXF were assessed between April 2021 and April 2022. YYSXF was administered alongside CHOP chemotherapy (Test group, *n* = 5) to monitor blood cell parameter reduction, and compared with canines receiving only CHOP chemotherapy (Control group, *n* = 6) to evaluate YYSXF’s efficacy.

**Results:**

YYSXF treatment improved the numbers of peripheral red blood cells (RBCs), white blood cells (WBCs), neutrophils (NEUTs), and platelets (PLTs), while reducing apoptosis and promoting cell cycle progression in bone marrow cells (BMCs) in myelosuppressed mice, however, validation in larger cohorts remains necessary. YYSXF also increased BMNC counts and the percentage of HSCs in BMCs, alleviating reductions in hematopoietic cell counts and fat vacuolation in the bone marrow. In the clinical phase, a decrease in complete blood count (CBC) indicators was observed after the eighth chemotherapy cycle in multicentric lymphoma canines, significantly delaying the onset of chemotherapy-induced reductions (*p* < 0.05) compared to the Control Group (third chemotherapy cycle).

**Discussion:**

These findings suggest the potential efficacy of YYSXF in supporting bone marrow hematopoiesis in mice, with further validation in canine models needed before clinical application.

## 1 Introduction

Lymphoma is one of the most common malignant neoplasms in canines, representing 83% of hematopoietic neoplasms and 7–24% of all tumors (1). Lymphomas are classified by anatomical location into multicentric, alimentary, and mediastinal types (2). Multicentric lymphomata accounts for about 80% of all cases and exhibits systemic and malignant characteristics, chemotherapy is the first-line as a treatment in clinical practice (2–4). The combination of cyclophosphamide (C), doxorubicin (H), vincristine (O), and prednisolone (P) (CHOP) is one of the most commonly recommended chemotherapy regimens for lymphoma (5). Although most patients respond well to CHOP therapy, it also affects normal cells, leading to a range of adverse effects and complications (6, 7), including gastrointestinal disturbances (14.8%) and myelotoxicity (20.0%) (8). In addition to fatigue, anemia, and gastrointestinal issues, bone marrow suppression is one of the most common complications of chemotherapy (9). The primary clinical symptom of myelosuppression is a reduction in the number of peripheral blood cells, particularly of RBCs, WBCs, NEUTs and PLT (10). Veterinary medicine refers to the “General Terminology Standard for Adverse Events” issued by the U. S. Department of Health in human medicine and has formulated the “Veterinary Tumor Cooperative Group-General Terminology Standard for Adverse Events (VCOG-CTCAE).” This system divides bone marrow suppression and blood toxicity into five grades to more accurately reflect clinical severity based on blood cell counts (11).

Bone marrow suppression can impair immune function; however, the associated infection risk depends on factors such as chemotherapy dose intensity, treatment schedule, and drug combination. In certain chemotherapy regimens, critical components of adaptive immunity—such as T-cell-mediated responses and antibody production—may be preserved, thus reducing the overall infection risk (12). However, the lack of standardized protocols for managing myelosuppression in veterinary medicine may prolong and intensify this adverse effect, potentially compromising antitumor efficacy. Current strategies primarily involve modifying the chemotherapy regimen, reducing medication doses, or delaying treatment (13). Antimicrobial prophylaxis and consideration of hospitalization are typically guided by ANC thresholds, with recent evidence supporting a cut-off of < 0.75 × 10^9^/L as safe and effective in reducing unnecessary antibiotic use while minimizing infection risk (14). As noted in previous studies, although myelosuppression is a clinically important complication requiring monitoring, severe cases necessitating hospitalization are relatively rare (15). Severe adverse reactions can result in multiple negative consequences, including reduced quality of life, increased medical costs due to hospitalization, delayed or altered treatment plans, and diminished client willingness to continue therapy. Under these circumstances, these interventions disrupt the continuity of chemotherapy and negatively impact antitumor efficacy. Recombinant GM-CSF (rGM-CSF) products have been shown to reduce the severity of bone marrow suppression and help restore blood cell populations to normal levels (16). However, these products also have adverse effects, including bone and muscle toxicity, as well as limited availability and high costs (17). Therefore, safer, more reliable, and more cost-effective therapies are needed to prevent and manage chemotherapy-induced complications and adverse effects.

Numerous studies have shown that traditional Chinese medicine (TCM) interventions effectively reduce chemotherapy-related side effects and improve patients’ quality of life (7, 18). Combining TCM with chemotherapy may enhance therapeutic efficacy and potentially reduce the frequency of chemotherapy-related side effects (19–21). Similarly, integrating TCM with veterinary oncology is considered a promising strategy for alleviating chemotherapy-related adverse effects. Numerous preclinical and clinical studies have shown that *Shi Quan Da Bu* decoction (SQDBD, Juzentaiho-to, or TJ-48 in Japanese), first recorded in the “Prescriptions of People’s Welfare Pharmacy” during the Song Dynasty, may help reduce the complications and side effects associated with chemotherapy and radiotherapy (22). SQDBD, a traditional remedy for treating *Qi* and blood deficiency syndromes, is commonly used in clinical practice to alleviate chemotherapy-induced myelosuppression in cancer patients (20). SQDBD is effective in alleviating chemotherapy-induced hematotoxicity, significantly increasing WBC counts, NEUT counts, and hemoglobin levels in the peripheral blood of patients with breast carcinoma (23).

*Yang Yin Sheng Xue* formula (YYSXF), evaluated in this exploration, is an innovative version of the classic tonifying-*Qi*-blood famous formula of SQDBD for veterinary clinical use. According to a series of descriptions in Chinese medicine literature, the primary effects of YYSXF based on the SQDBD prescription are to strengthen the kidney and spleen, nourish *Qi* and the blood, improve hematologic and immune function. Its low cost, availability, and high efficiency make it widely used in clinical practice.

The cyclophosphamide-induced mouse bone marrow suppression model is commonly used to simulate chemotherapy-induced myelosuppression in clinical settings (24). Based on our previous clinical applications, we hypothesized that YYSXF has hematopoietic effects and can be used for the prevention and/or treatment of chemotherapy-induced myelosuppression. To test this hypothesis, the effects of YYSXF on cyclophosphamide-induced myelosuppression in mice were evaluated. The hematopoietic effects of YYSXF were assessed, including changes in peripheral blood cell and BMNC counts, the proportion of bone marrow HSCs, apoptosis percentage, cell cycle analysis of BMCs, and histopathological examination of bone marrow. In the clinical phase of the study, canines with lymphoma were treated with either the CHOP chemotherapy regimen alone or CHOP chemotherapy combined with YYSXF. The effect of YYSXF in alleviating chemotherapy-induced bone marrow toxicity was indirectly evaluated by comparing the time to the first complete blood count (CBC) index reduction and the frequency of total and graded decreases in CBC data during treatment with the two approaches. This comparison provides a reference for the application of YYSXF in preventing and managing chemotherapy-induced myelosuppression in canines.

## 2 Materials and methods

### 2.1 Reagents

Cyclophosphamide (Baxter Oncology GmbH Co., Ltd.); YYSXF (with defined proportions and underwent batch consistency checks): superfine powder of traditional Chinese herbs (China Anguo city Xinhui Chinese Herbal Medicine Co., Ltd.), consists of fourteen different components—*Astragalus membranaceus* (Fisch.) Bunge, *Codonopsis pilosula* (Franch.) Nannf., *Dioscorea polystachya* Turczaninow, *Paeonia lactiflora* Pall., *Cuscuta chinensis* Lam., *Angelica sinensis* (Oliv.) Diels, *Pleuropterus multiflorus* (Thunb.) Nakai, *Ligustrum lucidum* Ait., *Millettia dielsiana* Harms, *Dolichos Lablab* L., *Wolfiporia cocos* (F. A. Wolf) Ryvarden Gilb., *Gallus gallus domesticus* Brisson, *Crataegus pinnatifida* Bunge, and *Hordeum vulgare* L., these constituents mixed at a weight ratio of 3:2:2:2:2:1:1:1:1:1:1:1:1:1; rGM-CSF (Beijing Zhongke Baike biology Co., Ltd.); RPMI 1640 culture medium, 1 × PBS, annexin V-FITC apoptosis detection kit, PI/RNase and JYBL-I decalcifying solution [Solarbio Science & Technology (Beijing) Co., Ltd.]; absolute ethyl alcohol and 4% paraformaldehyde fix solution (Beijing Aipu Ruisheng Technology Co., Ltd.); 3% acetic acid with methylene blue, FITC mouse lineage cocktail with isotype control, APC anti-mouse Ly-6A/E (Sca-1) antibody, and PE anti-mouse CD117 (c-kit) recombinant antibody (Beijing Annolun Biology Science and Technology Co., Ltd.); PI/RNase staining solution (Thermo Fisher Scientific [Beijing] Co.); vincristine (1 mg/vial, Guangdong Lingnan Pharmaceutical Co., Ltd.); doxorubicin (10 mg/vial, Shanxi Pude Pharmaceutical Co., Ltd.); and prednisolone (5 mg/tablet, Tianjin Lisheng Pharmaceutical Co., Ltd).

### 2.2 Experimental animals

A total of 36 C57BL/6 male, SPF-grade mice, aged 6 weeks (18 ~ 22 g), were acquired from Beijing Vital River Technology Co., Ltd. All mice were reared at a temperature of 21 ~ 24°C, 40% ~ 60% humidity, and continuous light/dark cycle. The mouse experiments were conducted at China Agricultural University Clinical Veterinary Department Laboratory.

### 2.3 Preparation of YYSXF turbid liquid

YYSXF is in the form of an ultra-fine powder that must be formulated into an herbal liquid for use. To prepare the herbal liquid, 50 g of ultra-micro powder of YYSXF was weighed into a beaker, and 500 mL of deionized water at 40°C was added. The mixture was stirred thoroughly using a magnetic stirrer and stored at 4°C. Prior to each use, the solution was warmed to room temperature in a water bath and then mixed thoroughly.

### 2.4 Mouse grouping, establishment of myelosuppression model, and drug administration

**Figure 1:**
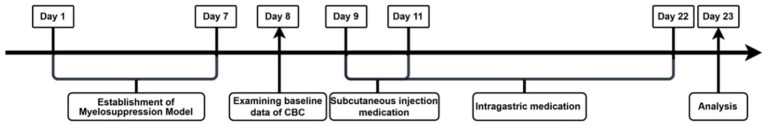
Schematic illustration of the experimental schedule. Mouse model phases of the study.

The experimental schedule is illustrated in Figure 1. 6 groups (Blank control, Model, Low-Y, Medium-Y, High-Y, and Positive control) were organized based on body weight, with six mice per group. Expect for the Blank control group, myelosuppression models in the other 5 groups were estabilished as previously described, with some modifications (24), by injecting intraperitoneally of cyclophosphamide (50 mg/kg) for 7 days. Normal saline (NS) was used instead of cyclophosphamide in the Blank control group. No placebo group was included in the present study due to ethical concerns in withholding potential treatment from sick animals. Baseline CBC data were collected for each mouse 24 h after the final injection, followed by the initiation of the assigned treatment. Table 1 summarizes the treatment dosage and duration for each group. The YYSXF dosage administered to dogs was derived from retrospective clinical data encompassing more than 100 canine lymphoma cases managed at China Agricultural University Veterinary Teaching Hospital, reflecting an empirically optimized therapeutic range.

**Table 1:** Subject groups and treatments in the mouse model experiment.

Group	Intragastric medication and duration	Subcutaneous injection medication and duration
Control	NS, Days from 1 to 14	NS, Days from 1 to 3
Model	NS, Days from 1 to 14	NS, Days from 1 to 3
Low-Y	0.3 g/kg YYSXF, Days from 1 to 14	NS, Days from 1 to 3
Medium-Y	0.6 g/kg YYSXF, Days from 1 to 14	NS, Days from 1 to 3
High-Y	1.2 g/kg YYSXF, Days from 1 to 14	NS, Days from 1 to 3
Positive	NS, Days from 1 to 14	50 μg/kg rGM-CSF, Days from 1 to 3

NS, saline solution.

### 2.5 Blood sample collection and CBC analysis

Blood samples were collected separately after modeling and drug administration. Approximately 100 μL of blood was obtained from each mouse via the submandibular vein and used for CBC analysis on an XN-1000 V blood analyzer (Sysmex Corp., Japan).

### 2.6 Preparation of bone marrow cell suspension

The mice were euthanized after blood samples were collected, and the femur and tibia were collected. The muscle tissue was removed from the bones, and the bone marrow cavity was repeatedly washed with RPMI 1640 medium. Clusters within the marrow suspension were collected and centrifuged to obtain the precipitate. Erythrocytes were lysed using an erythrocyte lysis buffer. A suspension of BMCs was prepared in PBS through repeated washing and centrifugation.

### 2.7 Determination of BMNCs number

The prepared bone marrow cell suspension was mixed, and 50 μL of the suspension was transferred into the centrifuge tubes. Then 450 μL of 3% acetic acid with methylene blue was added, and the mixture was thoroughly vortexed. A 10 μL aliquot of the mixture was pipetted onto the blood cell counting plate for manual counting.

### 2.8 Evaluation of the proportion of HSCs among BMCs

The cell suspension was stained by incubation with FITC anti–mouse Lineage Cocktail, PE anti–mouse CD117 (C-kit) recombinant, or APC anti–mouse-6A/E (SCA-1) at 4°C for 20 min away from light and next analyzed on a BD LSRFortessa flow cytometer (Becton, Dickinson and Company, USA).

### 2.9 Evaluation of the percentage of apoptotic of BMCs

Deionized water and binding buffer were added to the prepared bone marrow cell precipitate at a ratio of 1:9 (*v* / *v*). The cells were then analyzed using an Annexin V–FITC/PI apoptosis assay kit in accordance with the protocol, and the mixture was incubated away from light for 10 min before flow cytometry analysis.

### 2.10 Evaluation of cell cycle in BMCs

The cell suspension was stained by incubation with PI/RNase at 4°C for 20 min away from light and next analyzed on a BD LSRFortessa flow cytometer. For each group, 10,000 cells were consistently analyzed to ensure robustness and comparability of the data.

### 2.11 Histopathological observation

The sternum obtained from mice was fixed in 4% formaldehyde solution for histopathological examination. The sternum was then cut into tissue blocks of approximately 5 mm in length. The tissue blocks were washed with PBS and placed in a 10 × volume of JYBL-I decalcification solution for continuous decalcification over 2 days. After decalcification, the sternum tissue was processed for paraffin embedding. H. E. staining was used to assess structural changes in the bone marrow.

### 2.12 Clinical cases, groups, and drug administration

According to the established diagnostic criteria, inclusion criteria, exclusion criteria, and elimination and discontinuation criteria (Supplementary material S1), canines with multicentric lymphoma treated with CHOP chemotherapy and admitted to China Agricultural University Veterinary Teaching Hospital between April 2021 and April 2022 were enrolled in the study. Canines with multicentric lymphoma were diagnosed and classified according to World Health Organization (WHO) clinical staging standards, following physical examination, blood biochemical testing, diagnostic imaging, pathological lymph node cytology, and molecular biology analyses. The cell lineage of all lymphoma cases was confirmed using PCR for antigen receptor rearrangements (PARR). The Control group consisted of cases treated with CHOP chemotherapy alone, while the Test group included cases treated with both CHOP chemotherapy and oral YYSXF. The study covered the complete 19-weeks CHOP chemotherapy regimen. The dosages of YYSXF were shown in Table 2. The CHOP plan included 16 chemotherapy sessions, with a CBC test required before each session. Before the beginning of CHOP chemotherapy, the baseline characteristics (breed, age, weight, and disease stage) of the canines were statistically analyzed, and CBC analysis were recorded to confirm they were fell within the normal range. CBC parameters included RBCs, HCT, HGB, WBCs, and NEUTs. The evaluation indicators included the following: (1) time at which any one of the CBC observation indicators decreased below the limit of the normal range, and (2) total frequency and each frequency grade at which any one of the indicators decreased, the grading standard refers to the grading standard of Veterinary Oncology Cooperation Group-General Terminology Standard for Adverse Events (VCOG-CTCAE). The study was conducted at China Agricultural University Veterinary Teaching Hospital.

**Table 2:** Weight-dependent doses of YYSXF.

Weight (kg)	YYSXF dose
≤10	0.5 g, Bid, orally
10 ~ 20	1 g, Bid, orally
20 ~ 30	1.5 g, Bid, orally
30 ~ 40	2 g, Bid, orally
40 ~ 50	2.5 g, Bid, orally

Bid, twice a day.

### 2.13 Statistical analysis

Data were analyzed using GraphPad Prism 8.4 software. In the preclinical research section, results are presented as mean ± SD, based on a minimum of three independent experiments. Statistical analyses were conducted using one-way analysis of variance (ANOVA) test. *p* < 0.05 was considered statistically significant.

The Shapiro–Wilk test was performed on data of the clinical research section. Normally distributed data were described according to the mean ± SD, and differences between groups were evaluated using an independent t-test or one-way analysis of variance. Non-normally distributed data were described as the median, and differences between groups were evaluated using the Mann–Whitney *U* test.

## 3 Results

### 3.1 Evaluation of cyclophosphamide-induced myelosuppression in model mice

**Figure 2:**
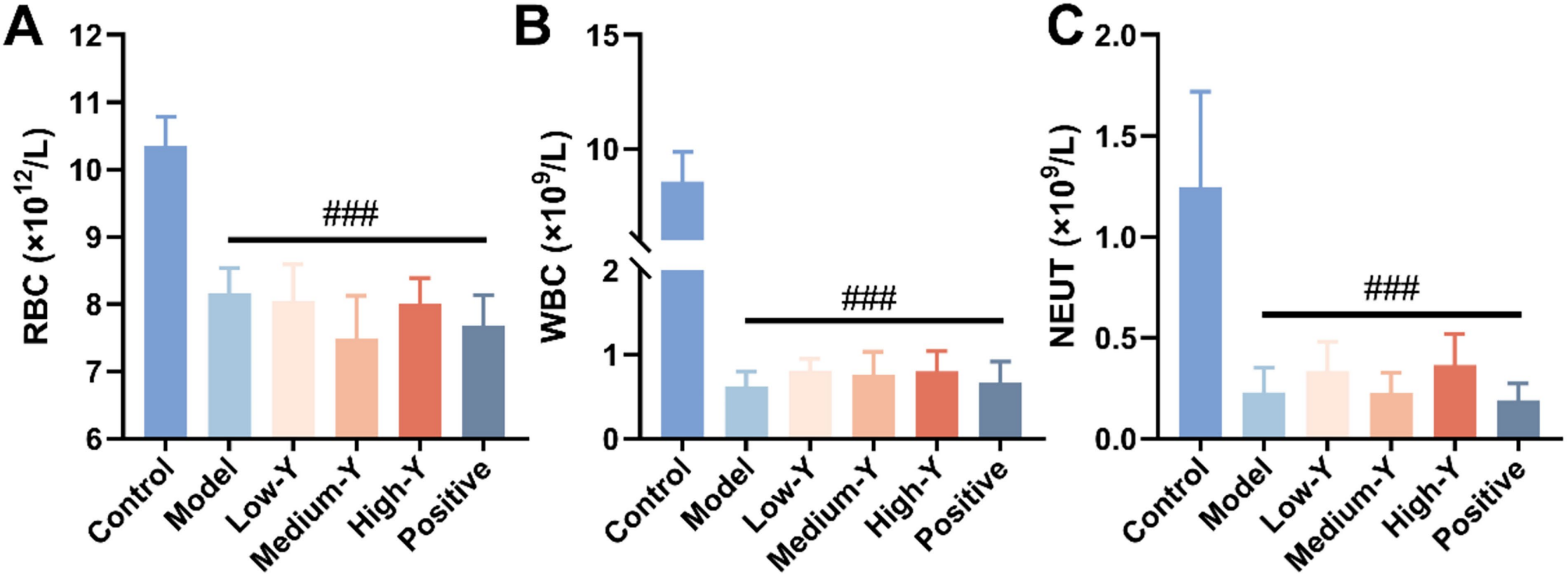
Evaluation of the mouse myelosuppression model (*n* = 6). **(A)** RBC. **(B)** WBC. **(C)** NEUT. ###: Compared with the control group, *p* < 0.001.

Compared with the control group, the RBC, WBC, and NEUT counts in other mouse myelosuppression model group were significantly decreased (*p* < 0.001), thus confirming that generation of the experimental myelosuppression mouse model was successful (Figures 2A–C).

### 3.2 Effect of YYSXF on peripheral blood cells

**Figure 3:**
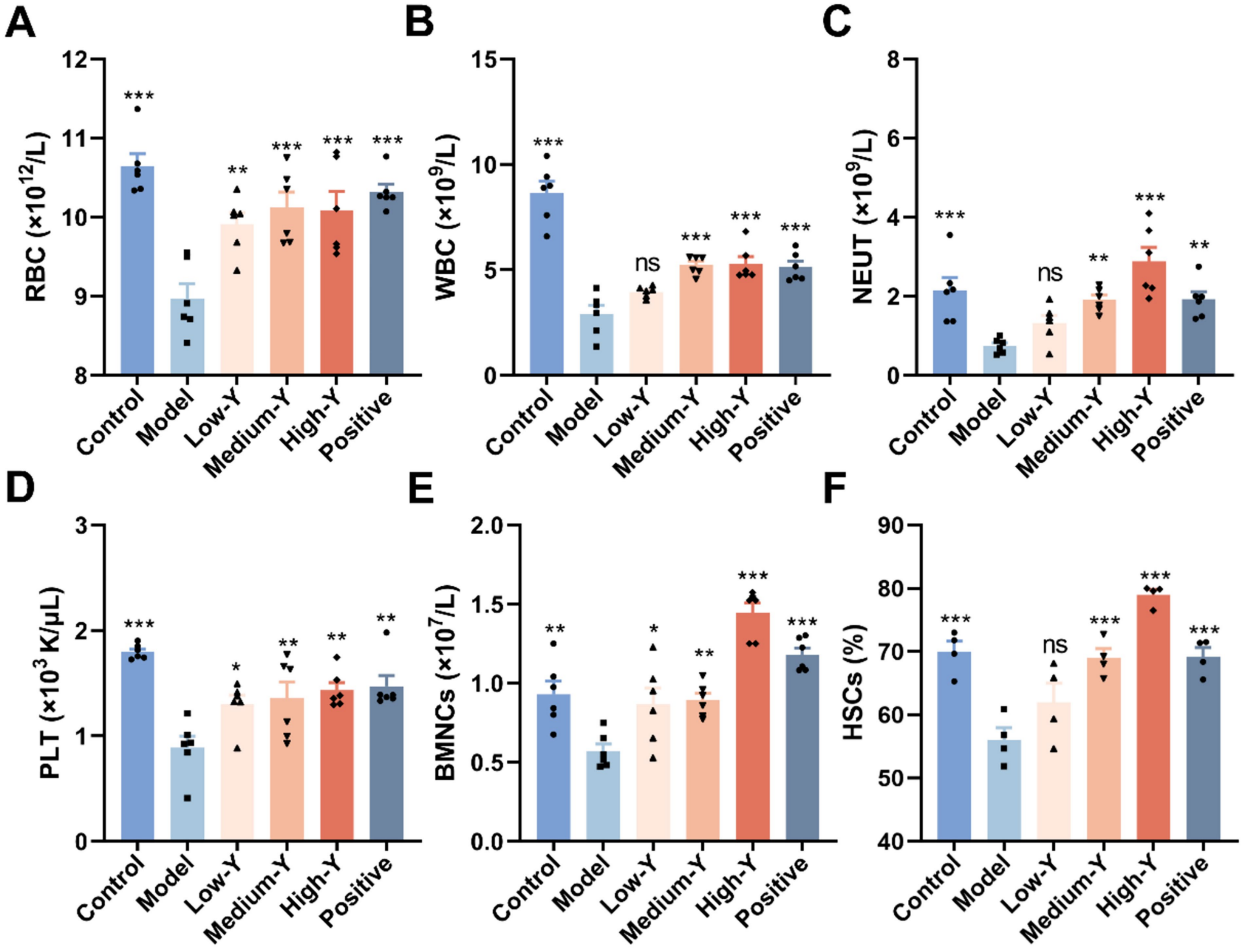
Effect of YYSXF on RBC **(A)**, WBC **(B)**, NEUT **(C)**, PLT **(D)**, BMNCs **(E)** counts and proportion of HSCs **(F)** in myelosuppression model mice (*n* = 4 ~ 6). *Compared with the model group, *p* < 0.05. **Compared with the model group, *p* < 0.01. ***Compared with the model group, *p* < 0.001.

RBC, WBC, NEUT and PLT counts for the six groups are shown in Figures 3A–D. After 14 days, the numbers of RBCs (8.97 ± 0.46 × 10^12^/L), WBCs (2.91 ± 1.01 × 10^9^/L), NEUTs (0.75 ± 0.19 × 10^9^/L) and PLT (0.89 ± 0.27 × 10^3^ K/μL) in the model group remained significantly lower than those in the control group (RBCs: 10.65 ± 0.38 × 10^12^/L, WBCs: 8.65 ± 1.35 × 10^9^/L, NEUTs: 2.15 ± 0.80 × 10^9^/L, PLT: 1.80 ± 0.07 × 103 K/μL) (*p* < 0.001), demonstrating that the myelosuppression model was stable for at least 14 days. The numbers of RBCs, WBCs, NEUTs and PLT in mice of all treatment groups were higher than those in the model group. Compared with the model group, the Low-Y (*p* < 0.01), Medium-Y (*p* < 0.001), High-Y (*p* < 0.001) and positive group (*p* < 0.001) was significantly higher in RBC count. The mice in the Medium-Y, High-Y and positive groups showed significantly elevated WBC counts, compared with the model group (*p* < 0.001). Figure 3C indicates that the NEUT in Medium-Y (*p* < 0.01), High-Y (*p* < 0.001) and positive group (*p* < 0.01) group were significantly increased, compared to that in the model group. The Low-Y, Medium-Y, High-Y and positive group elevated the number of PLT, compared with the model group (*p* < 0.05, *p* < 0.01, *p* < 0.01, *p* < 0.01).

### 3.3 Effect of YYSXF on number of BMNCs

The results of BMNC counts in the six groups of mice are shown in Figure 3E. The BMNCs in the model group were significantly decreased, compared to that in the normal control group (*p* < 0.01). In addition, the Low-Y group (0.87 ± 0.26 × 10^7^/L), the Medium-Y group (0.90 ± 0.10 × 10^7^/L), High-Y group (1.45 ± 0.15 × 10^7^/L), and positive control group (1.18 ± 0.10 × 10^7^/L) showed significant differences compared to the Model group (0.57 ± 0.11 × 10^7^/L) (Low-Y: *p* < 0.05, Medium-Y: *p* < 0.01, High-Y: *p* < 0.001, Positive: *p* < 0.001).

### 3.4 Effect of YYSXF on the percentage of bone marrow HSCs

The proportion of bone marrow HSCs in the myelosuppression model mouse was shown in Figure 3F. The percentage of HSCs in the model group (56.08 ± 3.79%) was significantly less than that in the control group (69.95 ± 3.39%) (*p* < 0.001). Compared with the model group, the percentages of YYSXF groups in all concentrations were higher: Low-Y (61.95 ± 6.16%), Medium-Y (69.00 ± 2.92%), and High-Y (79.00 ± 1.68%) (Medium-Y: *p* < 0.001; High-Y: *p* < 0.001). Additionally, the positive group (69.23 ± 2.81%) differed significantly from the Model group (*p* < 0.001).

### 3.5 Effect of YYSXF on percentage of apoptosis of BMCs

**Figure 4:**
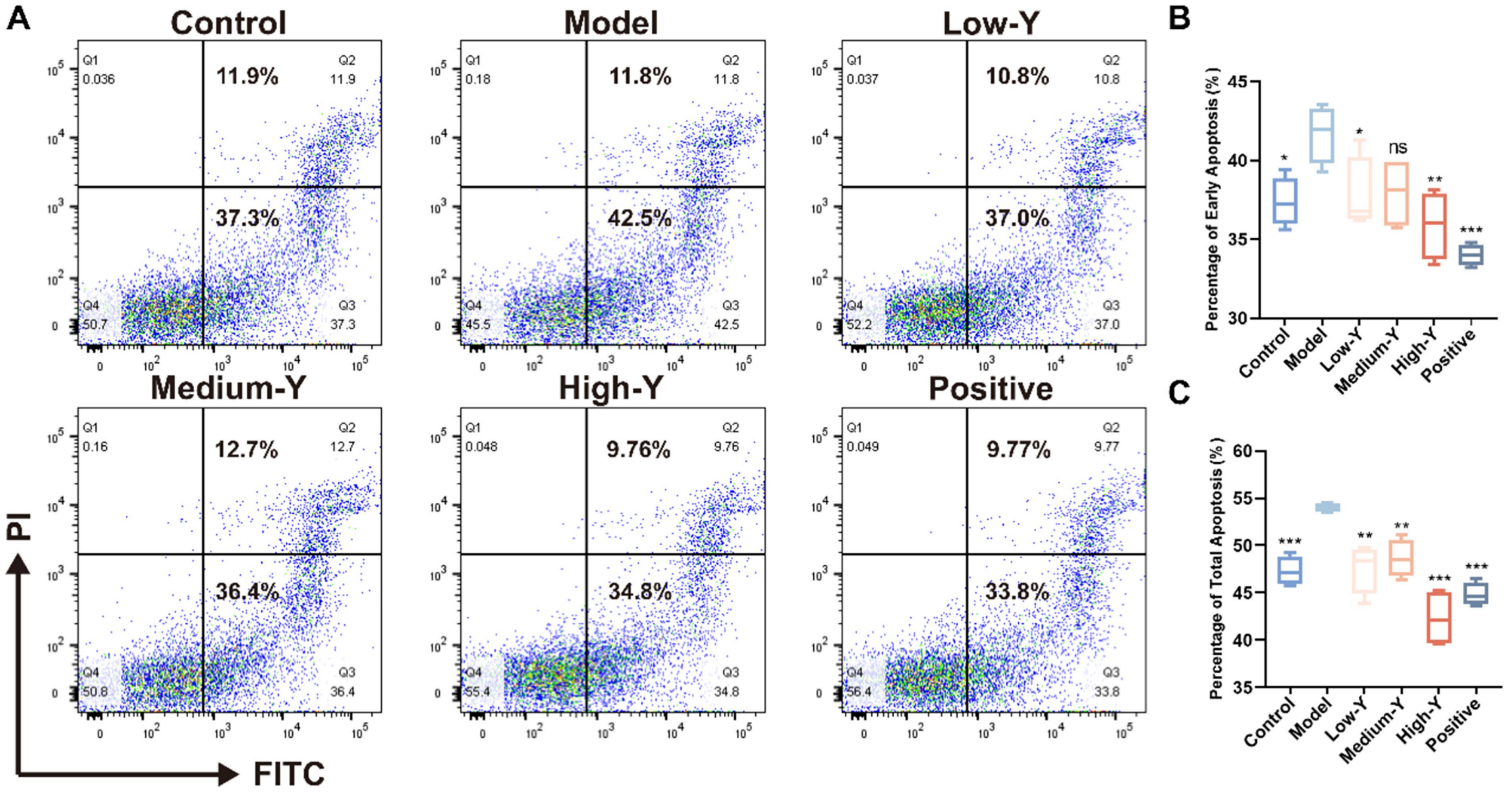
Effect of YYSXF on apoptosis in BMCs in a myelosuppression mouse model (*n* = 4). **(A)** Representative flow cytometry plots showing apoptosis in BMCs across treatment groups. **(B)** Quantification of early apoptosis (Q3 region). **(C)** Quantification of total apoptosis (Q2 + Q3 regions). *Compared with the model group, *p* < 0.05. **Compared with the model group, *p* < 0.01. ***Compared with the model group, *p* < 0.001.

The apoptosis percentage of mice BMCs was shown in Figures 4A–C. The proportion of early apoptotic cells in the model group (41.68 ± 1.80%) was significantly higher than that in the Blank control group (37.35 ± 1.56%) (*p* < 0.05). The Low-Y group (37.78 ± 2.37%), High-Y group (35.90 ± 2.18%) and positive control group (34.00 ± 0.67%) had significantly lower proportions compared to the model group (*p* < 0.05, *p* < 0.01, *p* < 0.001). The proportion of total apoptotic cells in the model group (53.98 ± 0.50%) was significantly higher than that in the control group (47.32 ± 1.48%) (*p* < 0.001). The proportion of total apoptotic cells in each treatment group was lower than that in the model group. The proportions of total apoptotic cells in the Low-Y group (47.62 ± 2.61%), Medium-Y group (48.60 ± 2.00%), High-Y group (42.32 ± 3.03%), and positive control group (44.81 ± 1.23%) were significantly lower than that in the model group (*p* < 0.01 *p* < 0.01, *p* < 0.01, *p* < 0.001, *p* < 0.001).

### 3.6 Effect of YYSXF on cell cycle of BMCs

**Figure 5:**
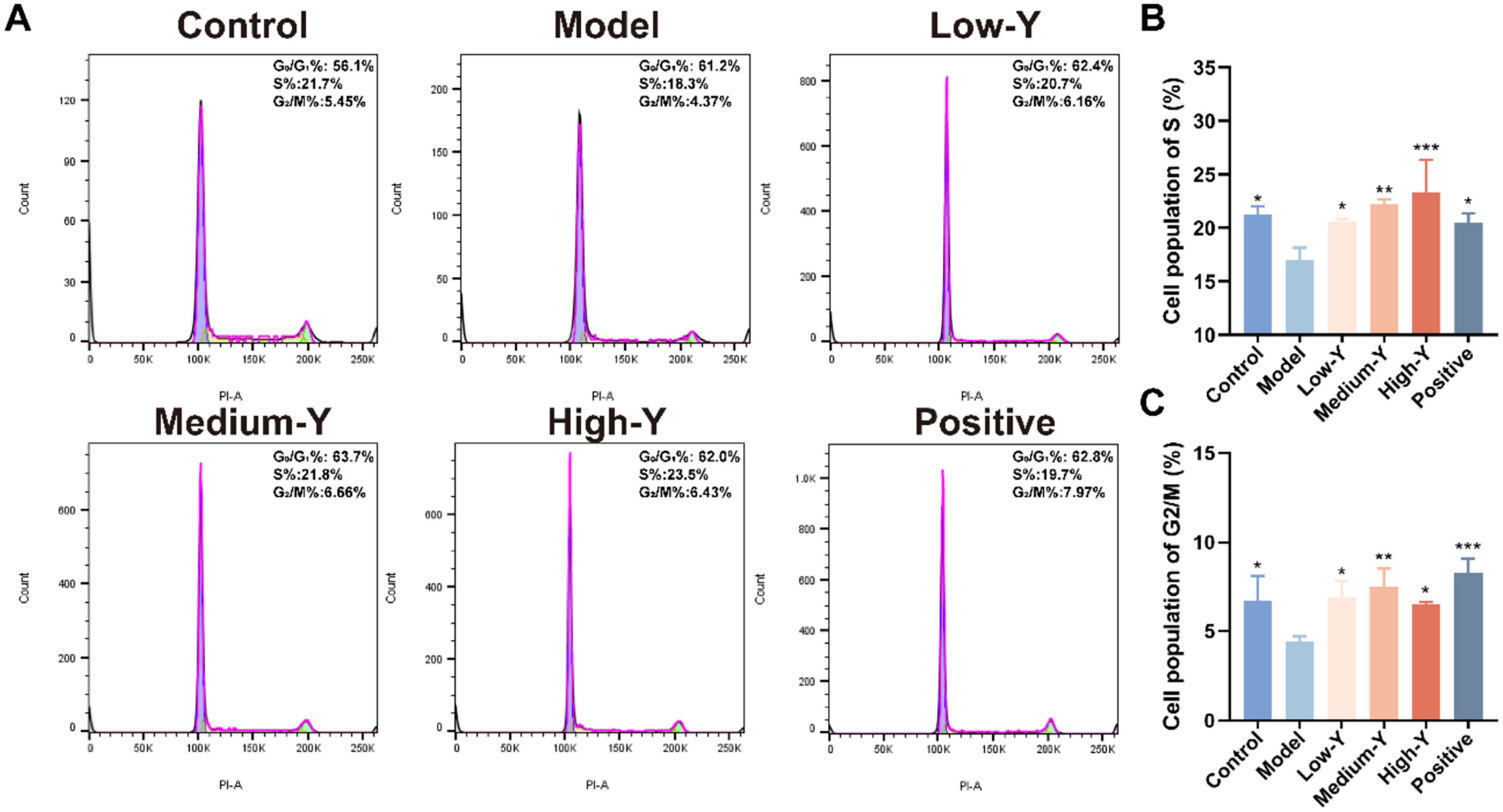
Effect of YYSXF on cell cycle among BMCs in myelosuppression model mice (*n* = 3). **(A)** Flow cytometry analysis of in BMCs after different treatments. **(B,C)** The percentages of cell population of S phase **(B)** and G2/M phase **(C)**. *Compared with the model group, *p* < 0.05. **Compared with the model group, *p* < 0.01. *** Compared with the model group, *p* < 0.001.

Furthermore, PI staining was conducted to explore the capacity of YYSXF on cell cycle progression. The proportions of cells in S phase and G2/M phase in mouse BMCs are shown in Figures 5A–C. The percentage of cells in S phase was significantly lower in the model group (17.03 ± 1.12%) compared to the blank control group (21.23 ± 0.81%) (*p* < 0.05). The Low-Y (20.60 ± 0.26%), Medium-Y (22.20 ± 0.46%), High-Y (23.33 ± 3.05%), and positive control groups (20.50 ± 0.85%) all showed significantly higher S phase percentages than the model group (*p* < 0.05, *p* < 0.01, *p* < 0.001, *p* < 0.05, respectively). The percentage of cells in the G2/M phase was significantly lower in the model group (4.41 ± 0.33%) compared to the control group (6.75 ± 1.36%) (*p* < 0.05). The percentages of cells in the G2/M phase were significantly higher in the Low-Y (6.90 ± 0.93%), Medium-Y (7.49 ± 1.04%), High-Y (6.52 ± 0.12%), and positive control (8.27 ± 0.81%) groups compared to the model group (*p* < 0.05, *p* < 0.01, *p* < 0.05, *p* < 0.001, respectively). These findings suggest that YYSXF facilitates the progression of bone marrow cells into the S and G2/M phases, thereby promoting cell cycle progression and potentially mitigating bone marrow suppression.

### 3.7 Bone marrow histopathological changes

**Figure 6:**
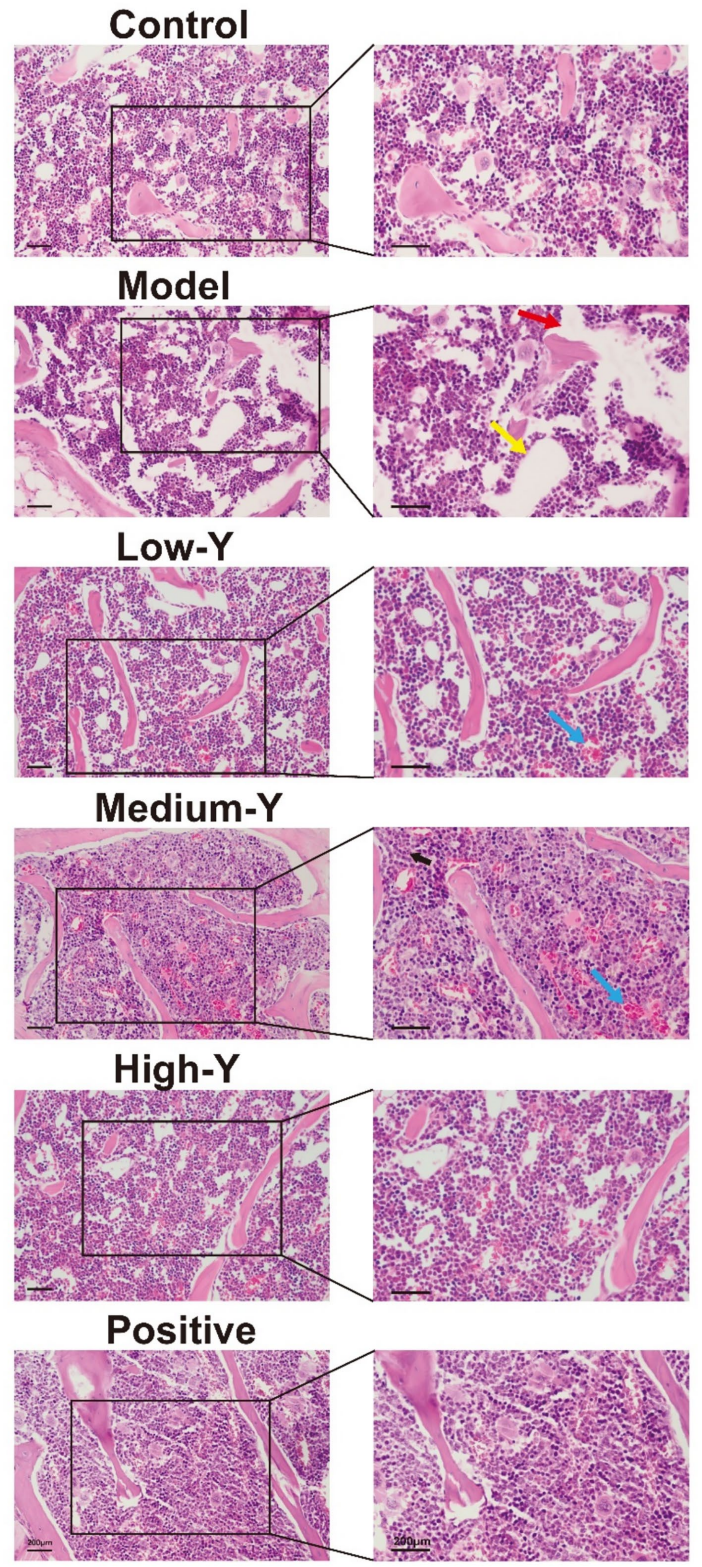
Histopathological images (40×) of the bone marrow of mice in the different groups after 14 days of treatment (Bar = 200 μm). Red arrow: hematopoietic cells. Yellow arrow: fat vacuolation. Blue arrow: capillary congestion. Black arrow: granulocyte.

Pathological sections of the bone marrow of model mice are shown in Figure 6. Compared to the control group, the bone marrow sinuses in the model group contained obviously fewer RBCs. In addition, the area of fat vacuolation increased (yellow arrow), while the density of hematopoietic cells decreased (red arrow). The bone marrow structure was improved in the Low-Y, Medium-Y, High-Y, and Positive control groups, with abundant hematopoietic cells, their morphology and structure are normal, and no obvious abnormality is found in trabecular bone, compared with the model group. Furthermore, a small amount of capillary congestion in the Low-Y and Medium-Y group (blue arrow).

### 3.8 Evaluation of the clinical application effect

#### 3.8.1 Signalment data of the clinical study

In the clinical study, 18 canines were included and assigned to either the Control group or Test group according to the owner’s decision, which resulted in 9 canines assigned to each group. According to the established diagnostic criteria, inclusion criteria, exclusion criteria, and elimination and discontinuation criteria, a total of 11 canines conducted and finished this clinical phase test (5 in the Test group and 6 in the Control group). The 11 canines that completed the study were of the following breeds: Labrador Retriever (*n* = 1, 9.1%), Border Shepherd (*n* = 1, 9.1%), Siberian Husky (*n* = 1, 9.1%), Chihuahua (*n* = 1, 9.1%), German Shepherd (*n* = 2, 18.2%), Corgi (*n* = 2, 18.2%), and Golden Retriever (*n* = 3, 27.3%). All lymphoma cases were confirmed to originate from B lymphocytes. In the Test group, three canines were classified as stage IV and two as stage V. In the Control group, five were at stage IV and one at stage III. The clinical distribution was stage III (*n* = 1, 9.1%), stage IV (*n* = 8, 72.7%), and stage V (*n* = 2, 18.2%). Supplementary Table S2 summarizes the statistical analysis of cases’ signalment data. The basic CBC values of the two groups of canines before chemotherapy were evaluated statistically (Supplementary Table S3). There was no significant difference between the two groups.

#### 3.8.2 Number of chemotherapies at the time of first CBC index decrease

The number of therapies was recorded when any one of the CBC indicators decreased below the normal range for the first time. The number of chemotherapies at the time of first CBC index decrease for each group of canines is shown in Table 3. The first decrease in a CBC index occurred after eight chemotherapies in Test group, compared with three chemotherapies in the Control group, indicating a significant delay in adverse events in the Test group (*p* < 0.05).

**Table 3:** Number of chemotherapies at time of first CBC index reduction (mean ± SD).

Group	Number of chemotherapy treatments at the time of first abnormal CBC index
Test group	8.40 ± 2.29*
Control group	3.00 ± 0.36

*p < 0.05, versus the Control group.

#### 3.8.3 Analysis of total frequency and each frequency grade related to CBC data

The total frequency of declines in RBC, HCT, HGB, WBC, and NEUT is shown in Table 4. The average total frequency in the Test group was lower than that in the Control group, although no significant difference was observed between the two groups. The frequencies of grade 1 and grade 2 declines in RBC, HCT, HGB, WBC, and NEUT in both groups of lymphoma canines are shown in Tables 5, 6. No grade 3 or higher decreases were observed in the Test group, while the average frequency of grade 3 decrease in NEUTs in the Control group was 0.16 ± 0.16 times. Additionally, no grade 4 or higher decreases were observed in the Control group.

**Table 4:** Total frequency of decreasing CBC indexes and five peripheral blood cell indexes during treatment (mean ± SD).

Group	CBC	RBCs	HCT	HGB	WBCs	NEUTs
Test group	6.20 ± 1.52	0.40 ± 0.40	0.80 ± 0.48	0.80 ± 0.48	1.60 ± 0.67	2.20 ± 0.58
Control group	16.00 ± 4.17	3.50 ± 1.54	2.66 ± 1.25	3.83 ± 1.42	2.50 ± 0.61	3.00 ± 1.29

**Table 5:** Frequency of grade 1 decreases in various peripheral blood cell parameters during treatment (mean ± SD).

Group	RBCs	HCT	HGB	WBCs	NEUTs
Test group	0.40 ± 0.40	0.80 ± 0.48	0.80 ± 0.48	1.20 ± 0.48	1.80 ± 0.58
Control group	3.00 ± 1.31	2.00 ± 0.77	3.16 ± 1.19	2.50 ± 0.61	2.33 ± 0.84

**Table 6:** Frequency of grade 2 decreases in various peripheral blood cell parameters during treatment (mean ± SD).

Group	RBCs	HCT	HGB	WBCs	NEUTs
Test group	/	/	/	0.20 ± 0.20	0.40 ± 0.24
Control group	0.50 ± 0.50	0.66 ± 0.66	0.66 ± 0.49	0.66 ± 0.49	0.50 ± 0.34

## 4 Discussion

Myelosuppression is a common complication of chemotherapy, in which bone marrow function is compromised, leading to fatigue, loss of appetite, dizziness, and variable effects on immune function. While canines with cancer may have pre-existing T-cell deficiencies, the impact of chemotherapy on immune function varies by protocol—for example, single-agent doxorubicin typically does not significantly reduce T-cell numbers, and even multi-agent CHOP chemotherapy, though decreasing B-cell counts, may preserve T-cell-mediated responses and antibody production, thus moderating the overall risk of infection (12). In severe cases, chemotherapy may need to be interrupted or delayed, which can negatively affect the efficacy of antitumor treatment (1). Currently, there is no appropriate management method for this condition in veterinary clinics. Our findings suggest that YYSXF may aid in the restoration of peripheral blood cell counts and bone marrow hematopoietic function following chemotherapy-induced myelosuppression; however, due to the non-randomized and unblinded nature of this study, further rigorously controlled trials are required to substantiate these preliminary results.

Analyses of hematopoietic parameters are routinely performed to monitor the adverse effects of chemotherapy. Results from the myelosuppression mouse model indicate that YYSXF, when administered within an appropriate concentration range, significantly promotes the proliferation of peripheral peripheral RBCs, WBCs, NEUTs, BMNCs, and PLTs. It also increases the proportion of HSCs, delays apoptosis in BMCs, and facilitates progression through the S and G2/M phases of the cell cycle, thereby demonstrating potent anti-myelosuppressive effects. Pathological analyses showed that YYSXF significantly reversed pathological changes in bone marrow tissue, increased the distribution of bone marrow hematopoietic cells, and simultaneously decreased the number of fat vacuoles. These results demonstrate that YYSXF delayed and reduced cyclophosphamide-induced blood and bone marrow toxicity, thereby improving hematopoietic function. However, potential risks associated with sustained stimulation of cell cycle progression—such as the theoretical possibility of enhancing proliferation in residual neoplastic cells—should not be overlooked. Therefore, comprehensive long-term safety evaluations are warranted to assess the effects of prolonged YYSXF administration on disease progression and overall therapeutic outcomes.

Several effective components of YYSXF have been investigated. The active component paeoniflorin, extracted from *Paeonia lactiflora* Pall., has been shown to reverse the decrease in WBCs in the peripheral blood of cyclophosphamide-induced myelosuppression model mice by day 11. It also significantly promotes the expression of hematopoiesis-related markers, including GM-CSF, G-CSF, and IL-3, providing notable protection against chemotherapy-induced myelosuppression (25, 26). 2,3,5,4′-tetrahydroxystilbene-2-O-*β*-D-glucoside (TSG), an active component from Pleuropterus multiflorus (Thunb.) Nakai, has been shown to slow the aging of HSCs and hematopoietic progenitor cells (HPCs), enhancing the regeneration of these cells by activating the energy receptor AMPK (27). Polysaccharide extracts from *Dioscorea polystachya* Turczaninow promote the recovery of bone marrow hematopoietic function by increasing the expression of PCNA and Survivin in bone marrow cells. This may be linked to the increased expression of MMP-2 and MMP-9, as well as the activation of proMMP-2 and proMMP-9 in the bone marrow hematopoietic microenvironment (28). Based on the above findings, it is speculated that YYSXF could increase peripheral blood cell production, aid in the recovery of early hematopoietic cells, and alleviate myelosuppression by regulating the expression of hematopoiesis-related cytokines and proteins, activating related receptors and pathways, and repairing the bone marrow hematopoietic microenvironment. Furthermore, future studies should focus on isolating and characterizing individual components of YYSXF to identify potential allergens and assess both their immunogenicity and therapeutic roles. It would also be valuable to evaluate modified or component-depleted formulations to determine whether the anti-myelosuppressive effects of YYSXF are retained.

Our clinical observations preliminary suggest that YYSXF may contribute to delaying the onset of chemotherapy-induced bone marrow suppression in dogs with lymphoma; however, due to the limited sample size, further large-scale, controlled studies are required to confirm this potential benefit. Currently, no clinical trials have been reported on the use of CHM for treating chemotherapy-induced myelosuppression in canines; however, several human clinical studies are available. Studies have shown that decoctions of *Astragalus membranaceus* (Fisch.) Bunge can reduce chemotherapy-related side effects, particularly nausea and vomiting, and decrease the incidence of chemotherapy-induced leucopenia in patients with bowel cancer (20, 29). Studies have shown that the TCM prescription Danggui Jixueteng decoction, composed of *Angelica sinensis* (Oliv.) Diels and *Millettia dielsiana* Harms, promotes the production of hematopoietic cytokines by activating the PI3K-Akt signaling pathway, thereby maintaining the balance of the hematopoietic microenvironment (30, 31). This suggests that using compatible herbs may promote the recovery of bone marrow hematopoietic function and reduce adverse effects in patients.

Our study results demonstrate that YYSXF can benefit canines with lymphoma undergoing chemotherapy. These results lay the foundation for developing a more secure and cost-effective integrated medical system, offering greater therapeutic impact and improving the quality of life for animals with cancer undergoing chemotherapy. Future research with larger sample sizes is needed to validate the therapeutic effects of varying doses of YYSXF and explore its additional clinical effects. Further validation in canine models is essential prior to clinical translation.

## 5 Conclusion

In this study, we aimed to identify a novel treatment strategy for canines with lymphoma undergoing chemotherapy. The experimental model findings suggest that YYSXF promotes peripheral blood cell formation and improves bone marrow hematologic function, thereby alleviating chemotherapy-induced bone marrow suppression. The clinical phase of the study indicated that YYSXF delays the initial chemotherapy-associated decrease in CBC indices, potentially improving the quality of life for canines with lymphoma undergoing chemotherapy. These findings suggest that YYSXF may be a promising treatment for chemotherapy-induced myelosuppression in canine lymphoma.

## Data Availability

The original contributions presented in the study are included in the article/Supplementary material, further inquiries can be directed to the corresponding authors.
