# Supplementary material for: Efficacy of Yang Yin Sheng Xue formula against canine lymphoma chemotherapy-induced myelosuppression
Source: Front Vet Sci. 2025 Aug 12;12:1635504. doi: 10.3389/fvets.2025.1635504 (PMC12378044; doi:10.3389/fvets.2025.1635504)
Supplement: Supplementary file 1 [file Table_1.docx]

**Supplementary materials**

**S1:**

**Diagnostic standards:**

After clinical basic examination, blood biochemical examination, imaging examination, pathological lymph node cytology and molecular biology examination, the dogs with multicentric lymphoma are diagnosed and divided into stages according to WHO clinical staging standards for multicentric lymphoma.

**Inclusion criteria:**

•Dogs with multicentric lymphoma that met the diagnostic criteria.

•Animal owners agreed to accept CHOP chemotherapy plan.

•KPS score ≥60, estimated survival time ≥ 4 months.

•No heart failure, liver and kidney function index did not exceed 1.25 times of the normal maximum value.

**Exclusion criteria:**

•Cases with other serious primary diseases involved cardiovascular, nervous, liver and kidney.

•Cases of using drugs related to stimulating hematopoietic function, regulating immune function or tonifying and nourishing herbs one week before the study.

**Cases with the following conditions were withdrawn from the trial:**

•Cases that failed to receive complete CHOP regimen chemotherapy.

•Cases of allergic or serious adverse reactions to formula YYSX.

•Cases that failed to follow the doctor's advice and take medication.

•Cases in which other drugs that stimulate hematopoiesis and tonify were used at the same time during the study.

•Cases that automatically withdrew from treatment.

**Table S2** **Summary of statistical analysis of signalment data (mean±SD).**

| Group | Age (years) | Body weight (kg) |
| --- | --- | --- |
| Test group (n = 5) | 7.20±3.27 | 31.18±10.04 |
| Control group (n = 6) | 7.33±2.73 | 19.53±9.2 |

**Table S3 The basic CBC values of two groups of canines before chemotherapy (mean±SD).**

| Group | RBCs  (×10^12^/L) | HCT  (%) | HGB  (g/L) | WBCs  (×10^9^/L) | NEUTs  (×10^9^/L) |
| --- | --- | --- | --- | --- | --- |
| Test group | 6.54±0.33 | 44.38±1.84 | 155.20±16.53 | 18.40±2.79 | 10.57±2.21 |
| Control group | 5.94±0.50 | 41.68±3.61 | 144.83±34.39 | 16.35±1.65 | 11.99±1.75 |
